# Association Between Life Purpose and Mortality Among US Adults Older Than 50 Years

**DOI:** 10.1001/jamanetworkopen.2019.4270

**Published:** 2019-05-24

**Authors:** Aliya Alimujiang, Ashley Wiensch, Jonathan Boss, Nancy L. Fleischer, Alison M. Mondul, Karen McLean, Bhramar Mukherjee, Celeste Leigh Pearce

**Affiliations:** 1 Department of Epidemiology, University of Michigan School of Public Health, Ann Arbor; 2 Department of Biostatistics, University of Michigan School of Public Health, Ann Arbor; 3 Department of Obstetrics and Gynecology, University of Michigan Health System, Ann Arbor

## Abstract

**Importance:**

A growing body of literature suggests that having a strong sense of purpose in life leads to improvements in both physical and mental health and enhances overall quality of life. There are interventions available to influence life purpose; thus, understanding the association of life purpose with mortality is critical.

**Objective:**

To evaluate whether an association exists between life purpose and all-cause or cause-specific mortality among older adults in the United States.

**Design, Setting, and Participants:**

The Health and Retirement Study (HRS) is a national cohort study of US adults older than 50 years. Adults between the ages of 51 to 61 were enrolled in the HRS, and their spouses or partners were enrolled regardless of age. Initially, individuals born between 1931 and 1941 were enrolled starting in 1992, but subsequent cohort enrichment was carried out. The present prospective cohort study sample was drawn from 8419 HRS participants who were older than 50 years and who had filled out a psychological questionnaire during the HRS 2006 interview period. Of these, 1142 nonresponders with incomplete life purpose data, 163 respondents with missing sample weights, 81 participants lost to follow-up, 1 participant with an incorrect survival time, and 47 participants with missing information on covariates were excluded. The final sample for analysis was 6985 individuals. Data analyses were conducted between June 5, 2018, and April 22, 2019.

**Exposures:**

Purpose in life was assessed for the 2006 interview period with a 7-item questionnaire from the modified Ryff and Keyes Scales of Psychological Well-being evaluation using a Likert scale ranging from 1 to 6, with higher scores indicating greater purpose in life; for all-cause and cause-specific mortality analyses, 5 categories of life purpose scores were used (1.00-2.99, 3.00-3.99, 4.00-4.99, 5.00-5.99, and 6.00).

**Main Outcomes and Measures:**

All-cause and cause-specific mortality were assessed between 2006 and 2010. Weighted Cox proportional hazards models were used to evaluate life purpose and mortality.

**Results:**

Of 6985 individuals included in the analysis, 4016 (57.5%) were women, the mean (SD) age of all participants was 68.6 (9.8) years, and the mean (SD) survival time for decedents was 31.21 (15.42) months (range, 1.00-71.00 months). Life purpose was significantly associated with all-cause mortality in the HRS (hazard ratio, 2.43; 95% CI, 1.57-3.75, comparing those in the lowest life purpose category with those in the highest life purpose category). Some significant cause-specific mortality associations with life purpose were also observed (heart, circulatory, and blood conditions: hazard ratio, 2.66; 95% CI, 1.62-4.38).

**Conclusions and Relevance:**

This study’s results indicated that stronger purpose in life was associated with decreased mortality. Purposeful living may have health benefits. Future research should focus on evaluating the association of life purpose interventions with health outcomes, including mortality. In addition, understanding potential biological mechanisms through which life purpose may influence health outcomes would be valuable.

## Key Points

**Question:**

Does an association exist between life purpose and all-cause or cause-specific mortality among people older than 50 years participating in the US Health and Retirement Study?

**Findings:**

This cohort study of 6985 adults showed that life purpose was significantly associated with all-cause mortality.

**Meaning:**

Life purpose is a modifiable risk factor and as such the role of interventions to improve life purpose should be evaluated for health outcomes, including mortality.

## Teaser

This cohort study assesses whether an association exists between life purpose and all-cause or cause-specific mortality among adults older than 50 years of age who participated in the US Health and Retirement Study.

## Introduction

A growing body of literature suggests that having a sense of purpose in life is associated with both physical and mental health and overall quality of life.^1,2^ Purposeful living has been defined in various ways. In general, purpose in life can be defined as “a self-organizing life aim that stimulates goals,”^1^ promotes healthy behaviors, and gives meaning to life.^3,4^

Individuals lacking purpose in life may feel hopeless and not have motivation to live an active and healthful life. Some studies report that those with a strong purpose in life engage in healthy behaviors^5^ and have better health outcomes for sleep disturbances,^6^ stroke incidence,^7^ poststroke quality of life,^8^ depression,^2^ and diabetes.^9^ The association between life purpose and overall mortality has also been explored previously, with Cohen and colleagues carrying out a meta-analysis of 9 prospective studies that examined this association.^1^ The studies used a variety of different measures of life purpose, with 6^10,11,12,13,14^ of the 9 studies^10,11,12,13,14,15,16,17^ relying on a single question.^1^ For example, in the studies conducted in Japan, participants are asked if they have *ikigai*, which is defined as “something to live for, the joy and goal of living.”^11,12^ The aforementioned meta-analysis showed that compared with individuals with lower life purpose, higher scores on study-specific life purpose scales were associated with survival (relative risk, 1.17; 95% CI, 0.75-0.91).^1^

The modified Ryff and Keyes Scales of Psychological Well-being assessment is the most comprehensive, validated scale for measuring purpose in life.^1,4,5,18,19,20,21^ Only 2 of the studies included in the aforementioned meta-analysis^1^ used this instrument, and both found associations indicating that individuals with stronger purpose in life had lower all-cause mortality.^15,17^ Furthermore, each of the studies included in the meta-analysis adjusted for some potentially confounding factors, but none comprehensively accounted for other psychological well-being constructs, such as depression, anxiety, cynical hostility, negative affect, optimism, positive affect, and social participation.

The Health and Retirement Study (HRS) is a US nationally representative, prospective cohort study that began in 1992 and has collected comprehensive data, including information on life purpose and other psychological well-being constructs. Life purpose was originally assessed in the HRS using an unvalidated measurement instrument designed by an HRS investigator; an initial study^16^ using this measurement found no association with all-cause mortality. However, in 2006, the HRS implemented the modified Ryff and Keyes Scales of Psychological Well-being^4^ assessment to measure life purpose. To our knowledge, there have been no previous analyses in HRS exploring the association between life purpose and cause-specific mortality using the modified Ryff and Keyes Scales of Psychological Well-being assessment. Therefore, we used the HRS data from 2006 through 2010 to examine whether purpose in life is associated with all-cause or cause-specific mortality among older adults. We were able to adjust for a large number of potential confounders, including other psychological well-being constructs that may affect the association between life purpose and mortality.

## Methods

### Study Design and Participants

Adults between the ages of 51 and 61 years were enrolled in the HRS, and their spouses or partners were enrolled, regardless of their age. Initially, individuals born between 1931 and 1941 were enrolled starting in 1992, but subsequent cohort enrichment was carried out. Details on HRS recruitment and data collection have been previously published^22,23^ and are presented in the eAppendix in the Supplement. The present study followed the Strengthening the Reporting of Observational Studies in Epidemiology (STROBE) reporting guideline for cohort studies with the exception of reporting 2 levels of confounder-adjusted estimates rather than including fully unadjusted estimates for the sake of brevity. This study used deidentified, publicly available data from the HRS; therefore, the Institutional Review Board at the University of Michigan exempted the present study from the need for review. Written informed consent was obtained from all HRS participants.

In 2006, half of the HRS participants were randomly selected to complete an additional psychological questionnaire; this was a leave-behind questionnaire that participants returned by mail and included the modified Ryff and Keyes Scales of Psychological Well-being assessment. Although the remaining half of the HRS participants were asked to fill out the additional psychological questionnaire in the next interview wave in 2008,^24^ the present study focuses only on the first half of participants who completed the psychological questionnaire in 2006. For the present study, this group was followed up through their 2010 visit.

The present sample was drawn from the 8419 participants who were older than 50 years and who had filled out the psychological questionnaire during the HRS 2006 wave. Of these 8419 participants, we excluded 1142 (nonresponders) with incomplete life purpose data, 163 with missing sample weights, 81 with loss to follow-up, 1 with an incorrect survival time, and 47 with missing information on marital status, educational level, smoking status, alcohol use, or physical activity. Our final sample for analysis was 6985 individuals (eFigure in the Supplement).

### Determination of Vital Status

The HRS assessed all-cause and cause-specific mortality between 2006 and 2010. Month and year and cause of death were recorded from a combination of 2 data sources, those reported by household members and those obtained through matching with the National Death Index. A 98.8% validation of deaths with essentially zero false-positives has previously been reported by HRS tracking studies.^25^ Survival time was calculated as the number of months from the date of 2006 interview to date of death. In total, 776 deaths occurred among our analytic cohort from 2006 to 2010. In addition to all-cause mortality, we further focused on the 4 most common causes of death in HRS: heart, circulatory, and blood conditions (n = 297); cancer and tumors (n = 208); respiratory tract system conditions (n = 108); and digestive tract system conditions (n = 86).

### Censoring

Participants who were still alive at the end of their last follow-up were censored. Censoring dates were determined as follows: (1) participants who were still alive and were followed up during the 2010 wave were censored at the date of the last interview from the 2010 interview wave (n = 5966); (2) participants who were followed up during the 2008 wave, but not the 2010 wave, were censored at the date of the last interview from the 2008 interview wave (n = 138); and (3) participants who were not followed up during the 2008 or 2010 wave but were interviewed during the 2012 wave were censored at the date of the last 2010 interview for any participant (n = 105).

### Assessment of Purpose in Life

Purpose in life was assessed in the 2006 interview wave by using a 7-item questionnaire from the modified Ryff and Keyes Scales of Psychological Well-being.^4^ The questionnaire used a Likert scale ranging from 1 (strongly disagree) to 6 (strongly agree). Ratings for items that were negatively worded were flipped such that higher scores indicated greater purpose in life on all questions. The mean of the individual scores was then calculated for each participant to create a final, composite life purpose score. For the all-cause mortality analysis, further categorization was conducted to create 5 categories of life purpose score for ease of interpretation (1.00-2.99, 3.00-3.99, 4.00-4.99, 5.00-5.99, and 6.00). For the cause-specific mortality analysis, some categories were combined owing to small numbers. Life purpose was also fit as a continuous variable and in quartiles, and the results were consistent with those observed with the aforementioned categories.

On the basis of the HRS recommendations for coding,^24^ participants who answered fewer than 3 questions among the 7 life purpose questions were considered to have incomplete life purpose data and were excluded (n = 1142 nonresponders) (eTable 2 in the Supplement). Because these nonresponders are part of the ongoing HRS cohort, we were able to examine the sociodemographic and other characteristics of this group to provide insight into potential generalizability and selection bias issues (eTable 2 in the Supplement). A total of 944 individuals would have been eligible for this analysis if they had completed the questionnaire (eFigure in the Supplement); eTable 2 in the Supplement provides a description of the demographic and relevant clinical characteristics of this group of individuals.

### Other Covariates

All covariates were assessed by self-report in 2006 and included sociodemographic characteristics, baseline health and behavioral characteristics, and psychological factors. Sociodemographic characteristics included age (50-54, 55-59, 60-64, 65-69, 70-74, 75-79, or ≥80 years old), sex (male or female), marital status (married, separated or divorced, widowed, or never married), race/ethnicity (non-Hispanic white, non-Hispanic and Hispanic black, Hispanic white, or other), and educational level (less than high school, high school degree, some college, college degree, or master’s or professional graduate degree). Baseline health behaviors/status included smoking status, functional status, frequency of physical activity, alcohol consumption, presence of 1 or more chronic health conditions, and body mass index^26^ (calculated as weight in kilograms divided by height in meters squared). Details on how these variables were modeled are available in the eAppendix in the Supplement. Psychological status included 4 negative psychological constructs (depression, anxiety, cynical hostility, and negative affect) and 3 positive psychological constructs (optimism, positive affect, and social participation). These constructs have been considered in previous analyses of life purpose data from the HRS and were thus considered here; they could be confounders or mediators. Each psychological construct consisted of multiple questions and was combined into a summary score based on the instructions from HRS for each of the 7 constructs.^24^ A description and the Cronbach α values for these 7 psychological constructs are given in eTable 1 in the Supplement. Each psychological construct was fit as a categorical variable based on quartiles.

### Statistical Analysis

Descriptive statistics for those who died and for those who were censored were calculated. Trend tests for continuous variables and heterogeneity for categorical variables were tested across groups using linear regression models and χ^2^ tests, respectively. Subsample specific weights provided by HRS were used in the present analysis to account for the HRS stratified, multistage area probability design.^27^ Weighted Cox proportional hazards models were fit to examine the association between life purpose score (categorical; see above) and all-cause and cause-specific mortality, using person-months as the underlying time metric. Two models were fit: model 1 was adjusted for sociodemographic factors as well as health characteristics at the 2006 interview, which included smoking status, physical activity, alcohol consumption, body mass index, presence of chronic health conditions, and functional status; and model 2 was adjusted for sociodemographic and health characteristics as well as psychological constructs. We estimated multivariate hazard ratios (HRs) and 95% CIs for all-cause and cause-specific mortality. In those analyses, we also took into account clustering within households given that individuals from the same household were invited to participate. In addition, we produced survival curves using the R package survminer (ggadjustedcurves) software to illustrate the association between life purpose and mortality. That curve was fully adjusted for sociodemographic and health characteristics and the psychological constructs.

In addition to our main analysis, we also conducted sensitivity analyses restricted to 1774 individuals who had no reported chronic health conditions (ie, cancer, heart disease, stroke, diabetes, lung disease, or high blood pressure) in 2006, and we treated 53 participants who died in the first year of follow-up as censored rather than as having the event. These analyses were undertaken to consider the potential for reverse causation as an explanation of our findings. We were concerned that being near to death leads to low life purpose or having a chronic illness leads to low life purpose. The goal of the sensitivity analysis was to determine whether there continued to be an association between life purpose and mortality in these population subsets. The power for these analyses was reduced because the sample size was smaller; thus, we evaluated only the trends in these data. All analyses were conducted from June 5, 2018, to April 22, 2019, using SAS, version 9.4 (SAS Institute Inc). A 2-sided *P* < .05 was considered statistically significant.

## Results

### Descriptive Analysis

In total, 6985 individuals were included in the analysis; 4016 participants (57.5%) were women, and the mean (SD) age of all participants was 68.6 (9.8) years. The mean survival time for decedents was 31.21 months (SD, 15.42 months; range, 1.00-71.00 months). Overall, individuals who were older, male, or not currently married were more likely to have died (Table 1). In addition, there was a greater percentage of individuals with less than a high school degree who died (283 of 776 [36.5%]) compared with that in the censored group (1378 of 6209 [22.2%]). Individuals who died were more likely to be a current or former smoker, be nonalcohol drinkers, be physically inactive, and have a lower functional status at the time of completing the life purpose questionnaire in 2006 (Table 1). The life purpose distribution is also provided in Table 1.

**Table 1.** Descriptive Characteristics of 6985 Health and Retirement Study Participants Included in This Analysis

Characteristic	Participants, No. (%)		*P* Value^a^
	No Event (n = 6209)	Death (n = 776)	
Age, y			
50-54	475 (7.7)	13 (1.7)	<.001
55-59	977 (15.7)	34 (4.4)	
60-64	976 (15.7)	56 (7.2)	
65-69	1266 (20.4)	121 (15.6)	
70-74	1052 (16.9)	116 (15.0)	
75-79	732 (11.8)	129 (16.6)	
≥80	731 (11.8)	307 (39.6)	
Sex			
Male	2588 (41.7)	381 (49.1)	<.001
Female	3621 (58.3)	395 (50.9)	
Marital status			
Married	4118 (66.3)	411 (53.0)	<.001
Separated or divorced	813 (13.1)	76 (9.8)	
Widowed	1110 (17.9)	266 (34.3)	
Never married	168 (2.7)	23 (3.0)	
Race/ethnicity			
Non-Hispanic white	4827 (77.7)	621 (80.0)	.12
Non-Hispanic and Hispanic black	780 (12.6)	96 (12.4)	
Hispanic white	327 (5.3)	40 (5.2)	
Other	275 (4.4)	19 (2.4)	
Educational level			
<High school	1378 (22.2)	283 (36.5)	<.001
High school	3145 (50.6)	368 (47.4)	
Some college	277 (4.5)	19 (2.4)	
College	838 (13.5)	59 (7.6)	
Master’s or professional degree	571 (9.2)	47 (6.1)	
Smoking status			
Never	2758 (44.4)	256 (33.0)	<.001
Current smoker	774 (12.5)	125 (16.1)	
Former smoker	2669 (43.0)	393 (50.6)	
Smoker but do not know whether current	8 (0.1)	2 (0.3)	
Consume alcohol, drinks/wk			
0	4079 (65.7)	588 (75.8)	<.001
1-2	994 (16.0)	100 (12.9)	
3-4	411 (6.6)	22 (2.8)	
5-6	208 (3.4)	13 (1.7)	
Every day	517 (8.3)	53 (6.8)	
Vigorous physical activity			
Every day	174 (2.8)	20 (2.6)	<.001
More than once a week	1338 (21.6)	76 (9.8)	
Once a week	502 (8.1)	30 (3.9)	
1-3 Times a month	423 (6.8)	28 (3.6)	
Hardly ever or never	3772 (60.8)	622 (80.2)	
Functional status score			
No.	6209	776	<.001
Mean (SD)	0.21 (0.66)	0.75 (1.26)	
Presence of chronic illness			
No	1695 (27.3)	79 (10.2)	<.001
Yes	4514 (72.7)	697 (89.8)	
BMI^b^			
≤18.50	191 (3.1)	62 (8.0)	<.001
18.51-24.99	1840 (29.6)	292 (37.6)	
25.00–29.99	2355 (37.9)	230 (29.6)	
≥30.00	1823 (29.4)	192 (24.7)	
Life purpose score			
No.	6209	776	<.001
Mean (SD)	4.57 (0.92)	4.05 (0.96)	
Life purpose score category			
1.00-2.99	217 (3.5)	83 (10.7)	<.001
3.00-3.99	1403 (22.6)	281 (36.2)	
4.00-4.99	2186 (35.2)	266 (34.3)	
5.00-5.99	1998 (32.2)	129 (16.6)	
6.00	405 (6.5)	17 (2.2)	

Abbreviation: BMI, body mass index (calculated as weight in kilograms divided by height in meters squared).

^a^ Obtained through χ^2^ test or *t* test.

^b^ Categorized according to the recommendations of the World Health Organization: below-normal weight (<18.5), normal range (18.5-24.9), overweight (25.0-29.9), and obesity (≥30.0).^26^

There were 1142 HRS participants who were eligible to complete the life purpose questionnaire in 2006 but either did not complete it at all (940 [82.3%]) or completed fewer than 3 questions (202 [17.7%]) (eTable 2 in the Supplement). The HRS participants who did not complete the life purpose questionnaire were no more likely to die than the individuals included in the analysis (121 of 944 [12.8%] compared with 776 of 6985 [11.1%]; *P* = .12). They were, however, more likely to be older, female, widowed, or African American; have less than a high school degree; and have weaker functional status at baseline compared with the participants who did complete the questionnaire. In addition, 191 of 1141 participants (16.7%) who did not complete the life purpose questionnaire were lost to follow-up between 2006 and 2010 compared with 81 of 7277 participants (11.1%) who did complete the questionnaire.

### Life Purpose and All-Cause Mortality

There was a significant association between life purpose and all-cause mortality (Table 2; Figure). This was true for both the model adjusted for sociodemographic and health characteristics (model 1 comparing individuals in the lowest life purpose group with individuals in the highest group: HR, 3.15; 95% CI, 2.07-4.77; *P* < .001 for trend) (Table 2) as well as model 2, which was additionally adjusted for psychological well-being constructs. In model 2, the HR was 2.43 for comparing individuals in the lowest life purpose group to individuals in the highest group (95% CI, 1.57-3.75; *P* < .001 for trend). Further adjustment for the psychological well-being constructs (model 2) attenuated the estimated HR in each category of life purpose score, but there remained a significant association between life purpose and mortality (Table 2; Figure).

**Table 2.** Data on Life Purpose and All-Cause Mortality Among 6985 Participants, 2006-2010 Health and Retirement Study^a^

Life Purpose Score Category	Hazard Ratio (95% CI)	
	Model 1^b^	Model 2^c^
1.00-2.99	3.15 (2.07-4.77)^d^	2.43 (1.57-3.75)^d^
3.00-3.99	2.13 (1.41-3.21)^d^	1.72 (1.13-2.62)^e^
4.00-4.99	1.90 (1.27-2.84)^e^	1.67 (1.12-2.49)^e^
5.00-5.99	1.30 (0.85-1.99)	1.26 (0.82-1.93)
6.00	1 [Reference]	1 [Reference]
*P* value for trend	<.001	<.001

^a^ All Cox proportional hazard models were weighted.

^b^ Cox proportional hazards model analysis results after adjusting for age, sex, educational level, race/ethnicity, marital status, smoking status, frequency of physical activity, alcohol consumption, body mass index, functional status, and presence of 1 or more chronic health conditions.

^c^ Cox proportional hazards model analysis results after adjusting for age, sex, educational level, race/ethnicity, marital status, functional status, smoking status, frequency of physical activity, alcohol consumption, body mass index, presence of 1 or more chronic health conditions, depression, anxiety, cynical hostility, negative affect optimism, positive affect, and social participation.

^d^ *P* < .001

^e^ *P* < .05.

**Figure.**
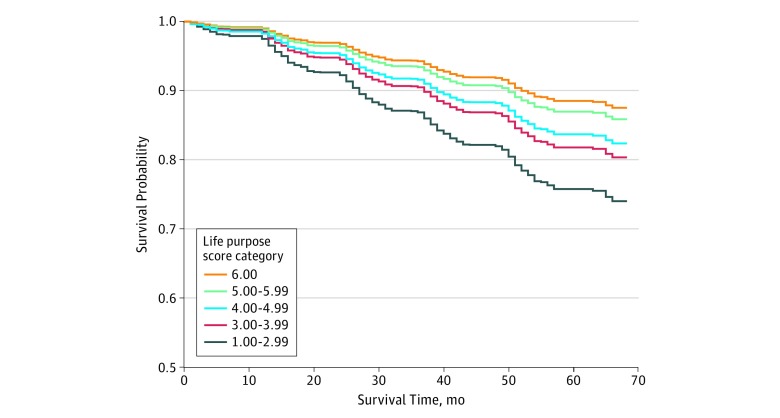
Survival Curves Illustrating the Association Between Life Purpose and Mortality Survival curves are adjusted for age, sex, educational level, race/ethnicity, marital status, smoking status, frequency of physical activity, alcohol consumption, body mass index, functional status, 1 or more chronic health conditions, depression, anxiety, cynical hostility, negative affect optimism, positive affect, and social participation. Adjusted survival curves are constructed by first generating a pseudopopulation based on study population characteristics. For each life purpose score category, a survival curve is estimated for this pseudopopulation based on the fitted Cox proportional hazards model presented in Table 2 assuming that all participants fall into that specific life purpose score category. This figure illustrates the relationship between life purpose and mortality on a covariate balanced pseudopopulation, with those in the lowest category (1.00-2.99) having significantly worse survival than those in the highest life purpose category (6.00).

We carried out sensitivity analyses to explore the potential of reverse causation as an explanation for the findings. After censoring participants who died during the first year of follow-up, the association between life purpose and risk of death was similar although modestly attenuated (highest vs lowest life purpose score: HR, 2.24; 95% CI, 1.44-3.50; model 4) (Table 3). Similarly, after excluding participants with a chronic health condition at baseline, there was still an association between life purpose and mortality (models 5 and 6) (Table 3).

**Table 3.** Sensitivity Analysis for All-Cause Mortality, 2006-2010 Health and Retirement Study

Life Purpose Score Category	Hazard Ratio (95% CI)			
	First Year Death Exclusion (n = 6985) (723 Events)^a^		Chronic Disease–Free at Baseline (n = 1774) (79 Events)^b^	
	Model 3^c^	Model 4^d^	Model 5^e^	Model 6^f^
1.00-2.99	2.85 (1.85-4.33)^g^	2.24 (1.44-3.50)^g^	2.22 (1.10-4.85)^g^	1.41 (0.59-3.39)
3.00-3.99	1.96 (1.30-2.96)^g^	1.61 (1.06-2.45)^g^		
4.00-4.99	1.78 (1.20-2.64)^g^	1.58 (1.06-2.34)^g^	2.12 (0.94-4.79)	1.76 (0.82-3.77)
5.00-5.99	1.29 (0.85-1.96)	1.24 (0.81-1.90)	1 [Reference]	1 [Reference]
6.00	1 [Reference]	1 [Reference]		
*P* value for trend	<.001	.002	.03	.55

^a^ Sensitivity analysis was conducted by censoring those who died in first year of follow-up (2006). All Cox proportional hazard models were weighted.

^b^ Sensitivity analysis was conducted by excluding those with chronic disease at baseline. All Cox proportional hazard models were weighted.

^c^ Cox proportional hazards model analysis after adjusting for age, sex, educational level, race/ethnicity, marital status, smoking status, frequency of physical activity, alcohol consumption, body mass index, functional status, and 1 or more chronic health conditions.

^d^ Cox proportional hazards model analysis after adjusting for age, sex, educational level, race/ethnicity, marital status, smoking status, frequency of physical activity, alcohol consumption, body mass index, functional status, 1 or more chronic health conditions, depression, anxiety, cynical hostility, and negative affect optimism, positive affect, and social participation.

^e^ Cox proportional hazards model analysis after adjusting for age, sex, educational level, race/ethnicity, marital status, smoking status, frequency of physical activity, alcohol consumption, body mass index, and functional status.

^f^ Cox proportional hazards model analysis after adjusting for age, sex, educational level, race/ethnicity, marital status, smoking status, frequency of physical activity, alcohol consumption, body mass index, functional status, depression, anxiety, cynical hostility, negative affect optimism, positive affect, and social participation.

^g^ *P* < .05.

### Life Purpose and Cause-Specific Mortality

There was a significant association between life purpose and mortality attributed to heart, circulatory, and blood conditions when the lowest and highest life purpose categories were compared (HR, 2.66; 95% CI, 1.62-4.38; *P* = .02 for trend) and between life purpose and digestive tract system conditions when the lower life purpose categories and the highest category were compared (for life purpose scores 3.00-3.99: HR, 4.54; 95% CI, 1.77-11.66; for life purpose scores 4.00-4.99: HR, 3.04; 95% CI, 1.34-6.89; *P* = .01 for trend) (Table 4). For cancer and tumors (HR, 1.16; 95% CI, 0.60-2.25; *P* = .95 for trend) and respiratory tract system conditions (HR, 1.83; 95% CI, 0.80-4.20; *P* = .40 for trend), however, no association with mortality was observed.

**Table 4.** Data on Cause-Specific Mortality Among 6985 Participants, 2006-2010 Health and Retirement Study^a^

Life Purpose Score Category	Hazard Ratio (95% CI)	
	Model 1^b^	Model 2^c^
Heart, circulatory, and blood conditions		
1.00-2.99	2.53 (1.69-3.78)^d^	2.66 (1.62-4.38)^d^
3.00-3.99	1.51 (0.92-2.49)	1.56 (0.93-2.62)
4.00-4.99	1.39 (0.87-2.22)	1.40 (0.87-2.27
5.00-6.00	1 [Reference]	1 [Reference]
*P* value for trend	<.001	.02
Cancer and tumors		
1.00-2.99	1.49 (0.79-2.80)	1.16 (0.60-2.25)
3.00-3.99	1.11 (0.66-1.86)	0.95 (0.53-1.69)
4.00-4.99	1.16 (0.77-1.74)	1.02 (0.66-1.56)
5.00-6.00	1 [Reference]	1 [Reference]
*P* value for trend	.43	.95
Respiratory tract system conditions		
1.00-2.99	3.43 (1.57-7.50)^d^	1.83 (0.80-4.20)
3.00-3.99	1.68 (0.99-2.86)	1.04 (0.56-1.93)
4.00-4.99	1.62 (1.03-2.54)^d^	1.24 (0.73-2.11)
5.00-6.00	1 [Reference]	1 [Reference]
*P* value for trend	.02	.40
Digestive tract system conditions		
1.00-2.99	2.68 (0.83-9.13)	2.05 (0.52-8.13)
3.00-3.99	5.95 (2.71-13.08)^e^	4.54 (1.77-11.66)^d^
4.00-4.99	3.52 (1.57-7.89)^d^	3.04 (1.34-6.89)^d^
5.00-6.00	1 [Reference]	1 [Reference]
*P* value for trend	<.001	.01

^a^ All Cox proportional hazard models were weighted.

^b^ Cox proportional hazards model analysis after adjusting for age, sex, educational level, race/ethnicity, marital status, smoking status, frequency of physical activity, alcohol consumption, body mass index, functional status, and 1 or more chronic conditions.

^c^ Cox proportional hazards model analysis after adjusting for age, sex, educational level, race/ethnicity, marital status, functional status, smoking status, frequency of physical activity, alcohol consumption, body mass index, 1 or more chronic health conditions. depression, anxiety, cynical hostility, negative affect, optimism, positive affect, and social participation.

^d^ *P* < .05.

^e^ *P* < .001.

## Discussion

Low purpose in life was significantly associated with death among 6985 older adults participating in the HRS. This finding was robust to adjustment for psychological well-being constructs and also showed a trend with decreasing life purpose. This finding is in agreement with previous literature that used either a different life purpose measurement tool, often asked only a single question, or adjusted for only a subset of the potential confounders for which we were able to control.^10,11,12,13,14,15,16,17^

There are several possible mechanisms through which life purpose might potentially be associated with mortality. Fredrickson and colleagues^28^ showed that stronger well-being, of which purposeful living is a component, was associated with decreased expression of proinflammatory genes. In another small study, stronger purpose in life was associated with lower cortisol levels and lower levels of proinflammatory cytokines.^19^ There is evidence to suggest that elevated levels of inflammatory markers, such as C-reactive protein,^29,30,31^ and cytokines, such as interleukin 6,^30,31^ are associated with increased mortality, and this represents one possible mechanism through which purpose in life influences mortality. However, to our knowledge, there have been no life purpose intervention studies that have then measured the impact on health outcomes (including mortality), cytokines, or other biomarkers.

The present results from the cause-specific mortality analysis were less clear. The association between mortality and life purpose was evident only for heart and blood diseases and digestive tract system diseases (Table 4). There is little published literature on cause-specific mortality and life purpose. Tanno and colleagues^12^ reported that the risk of cardiovascular mortality, but not coronary heart disease or cerebrovascular disease mortality, was significantly associated with life purpose among Japanese men. In addition, that study found a null association between life purpose and cancer as the cause of mortality, regardless of sex, which is in line with our null finding. Additional research on cause-specific mortality and life purpose is needed.

### Limitations

There are a number of method considerations with our analysis. Our study included a comprehensive analysis of psychological well-being constructs, including 4 negative psychological measures (depression, anxiety, cynical hostility, and negative affect) and 3 positive psychological measures (optimism, positive affect, and social participation). It is possible that some or all of these psychological well-being constructs are mediators; if this is the case, adjusting for these variables took the potential mediation into account. To address this issue, we presented results with or without adjustment for these measures (Table 2; model 1 vs model 2). Currently, more sophisticated methods for mediation analysis are not available because of the correlated nature of the psychological constructs and because this is a survival analysis. When such methods become available, it would be interesting to carry out a formal mediation analysis.

A potential concern with the present analysis is that life purpose at baseline may have been influenced by the presence of a chronic or life-threatening illness (reverse causation); however, this did not appear to be the case. We conducted sensitivity analyses in which all individuals who died during the first 2 years of follow-up were censored and included in a separate analysis, and we excluded all individuals who reported a chronic illness at baseline (Table 3). The results of these sensitivity analyses continued to suggest an association between life purpose and mortality (although not statistically significant given the major decrease in the number of events in the chronic conditions analysis), providing supporting evidence that reverse causation does not account for the association we observed.

Selective loss to follow-up does not appear to be a concern in the present study given that only 1% of the 7727 participants who completed the life purpose questionnaire were lost to follow-up. However, selection bias is a potential issue because among 1142 HRS participants in 2006 who were eligible to complete the life purpose questionnaire, 940 did not complete it at all, and 202 completed fewer than 3 questions. Those HRS participants were no more likely to die than the individuals included in the analysis (12.8% compared with 11.1%, *P* = .12), and we adjusted for the characteristics of those who were more likely to not complete the life purpose questionnaire; however, we cannot rule out selection bias.

## Conclusions

In summary, in the HRS, a stronger purpose in life was associated with lower all-cause mortality. There are a number of interventions that have been developed with the goal of improving life purpose. Intervention studies of volunteering,^32,33,34^ well-being therapy,^35^ and meditations^36^ among adults have shown improvements in purpose in life, quality of life, and various health outcomes. Mindfulness is one component of life purpose, and studies of this construct have been carried out among survivors of breast cancer.^37,38^ Those studies have shown promise in improving psychological well-being measures^37,38^ and quality of life,^38^ but associations with mortality have not been evaluated. There are also eHealth applications designed to influence life purpose (eg, Purposeful).^39,40^ Future research should focus on the mechanism of how life purpose may influence all-cause mortality and cause-specific mortality and on the appropriate timing of life purpose interventions in a diseased population. Ultimately, a randomized trial exploring life purpose interventions and disease outcomes, quality of life, and mortality are warranted.

## Notes

Supplement. **eAppendix.** Methods **eFigure.** 2006–2010 Health and Retirement Study Data Cleaning Flowchart **eTable 1.** Descriptive Statistics for Psychological Factors for the 2006–2010 Health and Retirement Study (N = 6,985) **eTable 2.** Descriptive Characteristics of the 944 Health and Retirement Study Participants With Incomplete Life Purpose Questionnaire Who Were Not Lost-to-Follow-up or Missing Covariate Data
