# Supplementary material for: Association Between Life Purpose and Mortality Among US Adults Older Than 50 Years
Source: JAMA Netw Open. 2019 May 24;2(5):e194270. doi: 10.1001/jamanetworkopen.2019.4270 (PMC6632139; doi:10.1001/jamanetworkopen.2019.4270)
Supplement: Supplement. — eAppendix. Methods eFigure. 2006–2010 Health and Retirement Study Data Cleaning Flowchart eTable 1. Descriptive Statistics for Psychological Factors for the 2006–2010 Health and Retirement Study (N = 6,985) eTable 2. Descriptive Characteristics of the 944 Health and Retirement Study Participants With Incomplete Life Purpose Questionnaire Who Were Not Lost-to-Follow-up or Missing Covariate Data [file jamanetwopen-2-e194270-s001.pdf]

Supplementary Online Content

Alimujiang A, Wiensch A, Boss J, et al. Association between life purpose and mortality among US adults older than 50 years. *JAMA Netw Open*. 2019;2(5):e194270. doi:10.1001/jamanetworkopen.2019.4270

eAppendix. Methods

eFigure. 2006–2010 Health and Retirement Study Data Cleaning Flowchart

eTable 1. Descriptive Statistics for Psychological Factors for the 2006–2010 Health and Retirement Study (N=6,985)

eTable 2. Descriptive Characteristics of the 944 Health and Retirement Study Participants With Incomplete Life Purpose Questionnaire Who Were Not Lost-to-Follow-up or Missing Covariate Data

This supplementary material has been provided by the authors to give readers additional information about their work.

© 2019 Alimujiang A et al. *JAMA Network Open*.

eAppendix. Methods

HRS Study Design

HRS cohort enrichment was carried out in 1993 (the Asset and Health Dynamics of the Oldest Old Cohort (AHEAD)), 1998 (Children of the Depression Cohort (CODA) and War Baby), 2004 (The Early Baby Boomers Cohort (EBB)) and 2010 (The Mid Baby Boomers Cohort (MBB) and Minority oversample) to enroll more birth cohorts (22). Face-to-face interviews are conducted every two years to collect information on participants' health, economics, demographics, and the retirement process; the data are publicly available (22, 23). HRS used a stratified, multistage area probability design, in which Blacks, Hispanics, and Floridians were oversampled (22, 23) and we accounted for the sampling weights in our analyses.

Covariate Coding

Smoking status was determined as never, current smoker, or former smoker. Individuals who reported being a smoker on previous questionnaires, but did not answer the question on the 2006 questionnaire, were placed into a separate "smoker, but current status unknown" category to prevent them from being excluded from the analysis. The functional status score was calculated by summation of five activities of daily living (bathing, eating, dressing, walking across a room, and getting in or out of bed). A higher score indicated weaker functioning (continuous). Physical activity was assessed based on the reported frequency of vigorous activity (such as running or jogging, swimming, cycling, aerobics or gym workout, tennis, or digging with a spade or shovel) and categorized as 1) every day; 2) more than once a week; 3) once a week; 4) one to three times a month; 5) hardly ever or never. Alcohol consumption was measured based on two questions "Do you ever drink any alcoholic beverages such as beer, wine, or liquor? and "If yes, in the last three months, on average, how many days per week have you had any alcohol to drink?" We further divided alcohol consumption into 1) non-drinkers 2) drink 1-2 days per week, 3) drink 3-4 days per week, 4) drink 5-6 days per week, and 5) drink everyday. BMI was calculated using self-reported weight and height, and categorized according to the recommendations of the World Health Organization (27): below-normal weight (<18.5 kg/m2), normal range (18.5–24.9 kg/m2), overweight (25.0–29.9 kg/m2), and obese (≥30.0 kg/m2).

© 2019 Alimujiang A et al. *JAMA Network Open*.

eFigure. 2006–2010 Health and Retirement Study Data Cleaning Flowchart

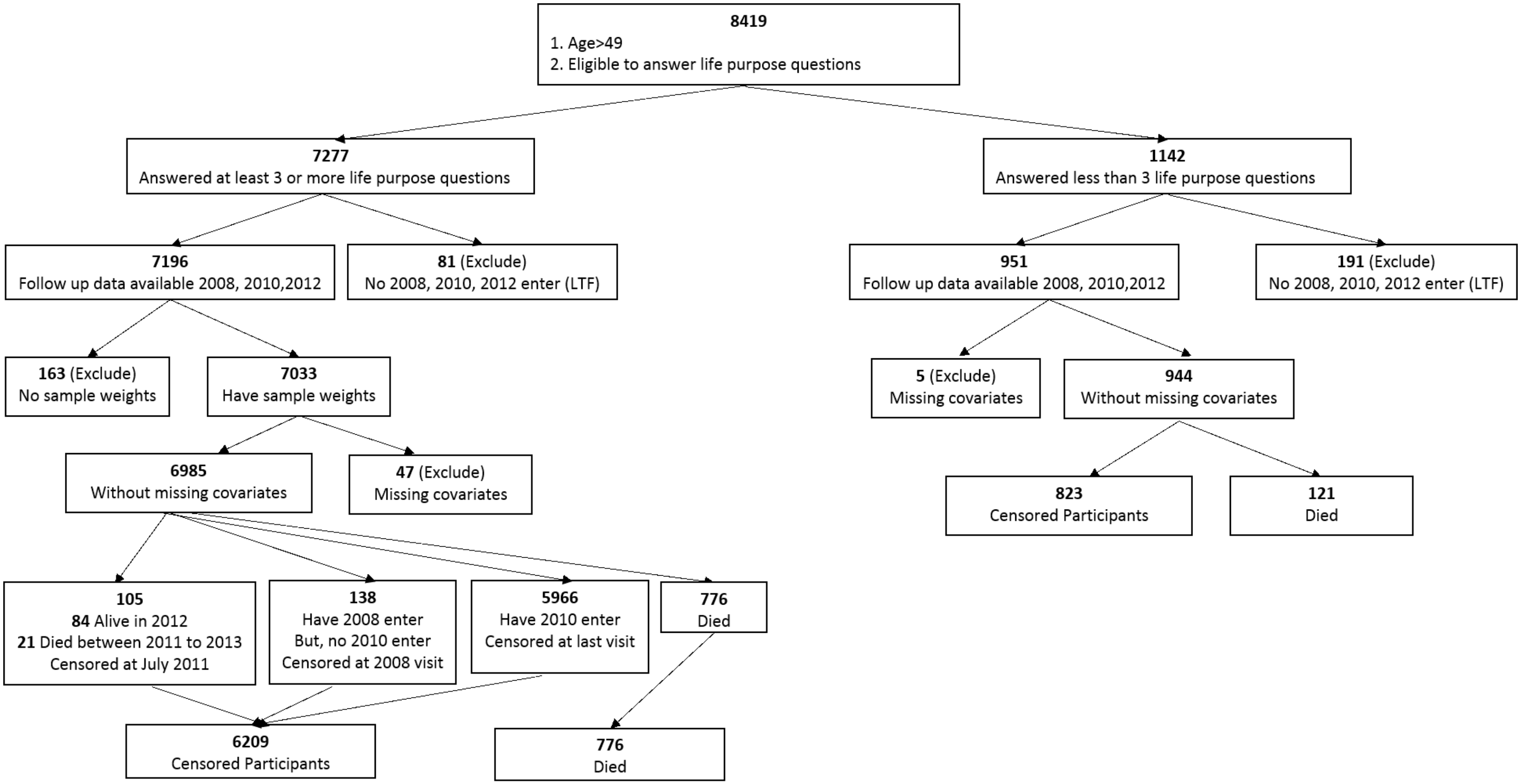

© 2019 Alimujiang A et al. *JAMA Network Open*.

eTable 1. Descriptive Statistics for Psychological Factors for the 2006–2010 Health and Retirement Study (N=6,985)

**Psychological covariates**	**No Event N=6209**			**Death N=776**			**Cronbach alphas[a]**
	Range	Mean	SD	Range	Mean	SD	
Negative							
Anxiety	1-4	1.54	0.56	1-4	1.72	0.64	0.81
Cynical hostility	1-6	2.94	1.13	1-6	3.10	1.18	0.79
Depression	0-8	1.45	1.9	0-8	2.25	2.12	0.54
Negative affect	1-5	1.60	0.63	1-5	1.87	0.77	
Positive							
Optimism	1-6	4.09	0.87	1-6	4.00	1.00	0.52
Positive affect	1-5	3.60	0.68	1-5	3.45	0.72	0.92
Social participation	0-8	4.19	1.66	0-8	3.07	1.69	0.87

[a] Standardized alpha coefficient provides information about how each variable reflects the reliability of the scale with standardized variables.

© 2019 Alimujiang A et al. *JAMA Network Open*.

eTable 2. Descriptive Characteristics of the 944 Health and Retirement Study Participants With Incomplete Life Purpose Questionnaire who were not lost-to-follow-up or missing covariate data (see eFigure).

	Total (N=944)		No Event (N=823)		Death (N=121)	
**Measure**	**N**	**Mean(SD)/%**	**N**	**Mean(SD)/%**	**N**	**Mean(SD)/%**
**Age**						
50-54	126	13.35%	125	15.19%	1	0.83%
55-59	148	15.68%	145	17.62%	3	2.48%
60-64	110	11.65%	106	12.88%	4	3.31%
65-69	143	15.15%	129	15.67%	14	11.57%
70-74	120	12.71%	103	12.52%	17	14.05%
75-79	84	8.90%	71	8.63%	13	10.74%
≥80	213	22.56%	144	17.50%	69	57.02%
**Sex**						
Male	397	42.06%	342	41.56%	55	45.45%
Female	547	57.94%	481	58.44%	66	54.55%
**Marital status**						
Married	503	53.28%	458	55.65%	45	37.19%
Separated/Divorced	143	15.15%	132	16.04%	11	9.09%
Widowed	266	28.18%	205	24.91%	61	50.41%
Never Married	32	3.39%	28	3.40%	4	3.31%
**Race/ethnicity**						
Non-Hispanic White	546	57.84%	469	56.99%	77	63.64%
Non-Hispanic and Hispanic Black	252	26.69%	222	26.97%	30	24.79%
Hispanic White	73	7.73%	66	8.02%	7	5.79%
Other	73	7.73%	66	8.02%	7	5.79%
**Education level**						
<High school	361	38.24%	309	37.55%	52	42.98%
High school	385	40.78%	330	40.10%	55	45.45%
Some College	34	3.60%	31	3.77%	3	2.48%
College	93	9.85%	86	10.45%	7	5.79%
Master or Professional degree	71	7.52%	67	8.14%	4	3.31%
**Smoking status**						
Never	401	42.48%	357	43.38%	44	36.36%
Current smoker	162	17.16%	146	17.74%	16	13.22%
Former smoker	377	39.94%	317	38.52%	60	49.59%
Smoker but don’t know whether current	4	0.42%	3	0.36%	1	0.83%
**Consume alcohol drinks/wk**						
0	660	69.92%	570	69.26%	90	74.38%
1-2	133	14.09%	124	15.07%	9	7.44%
3-4	56	5.93%	49	5.95%	7	5.79%
5-6	32	3.39%	30	3.65%	2	1.65%
Everyday	63	6.67%	50	6.08%	13	10.74%
**Vigorous physical activity**						
Every day	21	2.22%	20	2.43%	1	0.83%
More than once a week	153	16.21%	147	17.86%	6	4.96%
Once a week	57	6.04%	54	6.56%	3	2.48%
One to three times a month	36	3.81%	32	3.89%	4	3.31%
Hardly ever or never	677	71.72%	570	69.26%	107	88.43%
**Functional status score**	944	0.54(1.21)	823	0.41(1.03)	121	1.39(1.82)

© 2019 Alimujiang A et al. *JAMA Network Open*.

**Presence of Chronic Illness**						
No	243	25.74%	226	27.46%	17	14.05%
Yes	701	74.26%	597	72.54%	104	85.95%
**BMI[a]**						
≤18.50	58	6.14%	50	6.08%	8	6.61%
18.51-24.99	304	32.2%	249	30.26%	55	45.45%
25.00–29.99	330	34.96%	296	35.97%	34	28.10%
≥30.00	252	26.69%	228	27.70%	24	19.83%
**Life purpose score**	944	3.95(0.33)	823	3.95(0.33)	121	3.94(0.37)
**Life purpose score category**						
1.00-2.99	12	25.00%	10	23.81%	2	33.33%
3.00-3.99	9	18.75%	8	19.05%	1	16.67%
4.00-4.99	15	31.25%	14	33.33%	1	16.67%
5.00-5.99	8	16.67%	7	16.67%	1	16.67%
6.00	4	8.33%	3	7.14%	1	16.67%
Missing	896		781		115	

[a]. BMI as weight in kilograms divided by the square of the height in meters, then categorized according to the recommendations of the World Health Organization: below-normal weight (<18.5 kg/m^2), normal range (18.5–24.9 kg/m^2), overweight (25.0–29.9 kg/m^2), and obesity (≥30.0 kg/m^2)
[b]. P-values obtained through chi-square test or Student's t-test.

© 2019 Alimujiang A et al. *JAMA Network Open*.
